# Cardiomyocyte senescence and the potential therapeutic role of senolytics in the heart

**DOI:** 10.20517/jca.2024.06

**Published:** 2024-05-30

**Authors:** Peiyong Zhai, Junichi Sadoshima

**Affiliations:** Department of Cell Biology and Molecular Medicine, Cardiovascular Research Institute, Rutgers-New Jersey Medical School, Newark, NJ 07103, USA.

**Keywords:** Aging, senescence, senescence-associated secretory phenotype, senolysis

## Abstract

Cellular senescence in cardiomyocytes, characterized by cell cycle arrest, resistance to apoptosis, and the senescence-associated secretory phenotype, occurs during aging and in response to various stresses, such as hypoxia/reoxygenation, ischemia/reperfusion, myocardial infarction (MI), pressure overload, doxorubicin treatment, angiotensin II, diabetes, and thoracic irradiation. Senescence in the heart has both beneficial and detrimental effects. Premature senescence of myofibroblasts has salutary effects during MI and pressure overload. On the other hand, persistent activation of senescence in cardiomyocytes precipitates cardiac dysfunction and adverse remodeling through paracrine mechanisms during MI, myocardial ischemia/reperfusion, aging, and doxorubicin-induced cardiomyopathy. Given the adverse roles of senescence in many conditions, specific removal of senescent cells, i.e., senolysis, is of great interest. Senolysis can be achieved using senolytic drugs (such as Navitoclax, Dasatinib, and Quercetin), pharmacogenetic approaches (including INK-ATTAC and AP20187, p16–3MR and Ganciclovir, p16 ablation, and p16-LOX-ATTAC and Cre), and immunogenetic interventions (CAR T cells or senolytic vaccination). In order to enhance the specificity and decrease the off-target effects of senolytic approaches, investigation into the mechanisms through which cardiomyocytes develop and/or maintain the senescent state is needed.

## Graphical Abstract

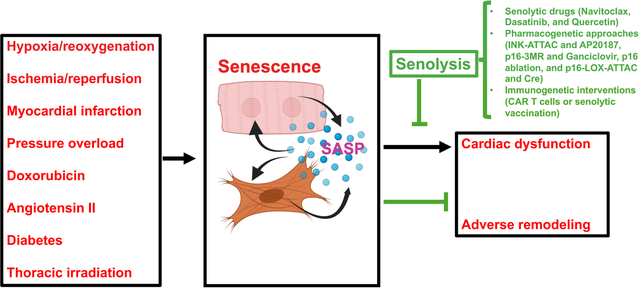

## INTRODUCTION

Aging is a critical risk factor for heart disease^[1]^. Remarkable increases in the aging population worldwide have been accompanied by an increased number of heart failure patients, imposing a significant socioeconomic burden on modern society^[2]^. Cellular senescence is a state of irreversible cell cycle arrest associated with aging and/or cellular stress, such as DNA damage, chromatin disruption, oncogene activation, reactive oxygen species (ROS), and mitochondrial dysfunction. Essential features of cellular senescence include activation of cell survival mechanisms and production of factors that induce inflammation and tissue remodeling, the latter of which is termed the senescence-associated secretory phenotype (SASP)^[3,4]^. Although senescence can be salutary in some instances, preventing tumorigenesis and facilitating tissue repair, persistent senescence precipitates the aging and inflammatory process in surrounding cells by paracrine mechanisms^[3,4]^. The development and spreading of senescent cells, especially senescent cardiomyocytes, in the heart may result in cardiac dysfunction and, eventually, heart failure^[5]^. This raises the possibility that selective elimination of senescent cells, termed senolysis, could prevent the development of cardiac dysfunction and aging in the heart^[6]^. In this mini-review, we will focus on cardiomyocyte senescence and the removal of senescent cardiomyocytes as a potential therapeutic strategy. We also highlight the current knowledge gaps in the field.

## SENESCENCE OF CARDIOMYOCYTES IN THE HEART

**Figure 1.**
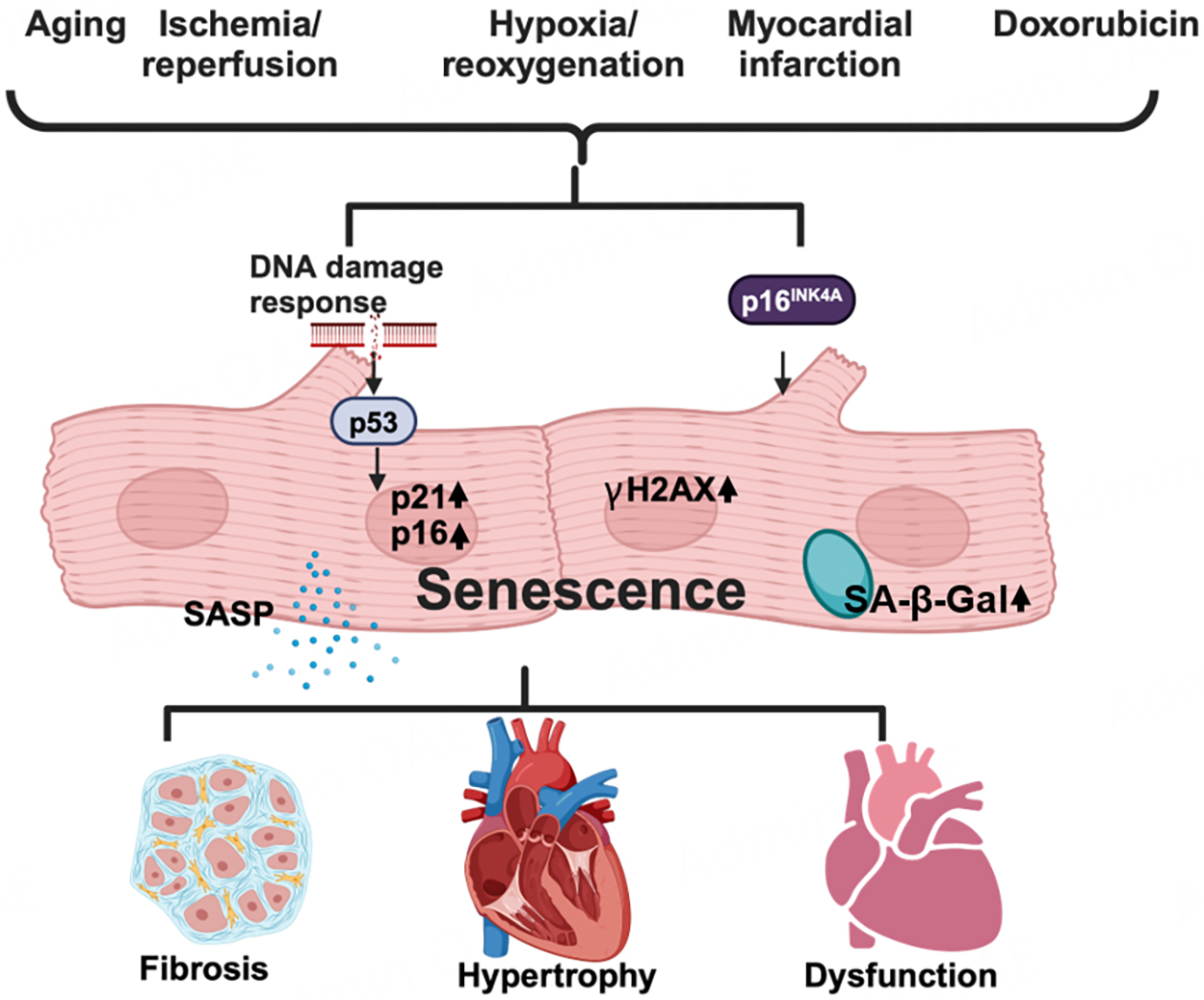
Cardiomyocyte senescence. Upstream stressors such as aging, ischemia, ischemia/reperfusion, and doxorubicin activate the DNA damage response/p53 and p16 signaling, which in turn leads to cardiomyocyte senescence. Senescent cardiomyocytes feature enlarged cell size, increased levels of p16, p21, and γH2AX, increased senescence-associated β-galactosidase (SA-β-gal) activity, and the senescence-associated secretory phenotype (SASP). The accumulation of senescent cardiomyocytes in the heart results in cardiac hypertrophy, fibrosis, and dysfunction. The figure was generated with BioRender.com.

Adult cardiomyocytes in humans are terminally differentiated, i.e., they are post-mitotic/rarely dividing. Thus, telomere shortening/attrition due to repetitive cell division, a major mechanism of senescence in proliferating cells, termed replicative senescence, may not occur in cardiomyocytes. However, length-independent telomere damage, which may be caused by ROS, induces senescence in terminally differentiated cardiomyocytes^[7]^. Accumulating lines of evidence suggest that cardiomyocytes develop senescence during aging^[7,8]^ and in response to stresses such as hypoxia/reoxygenation^[9]^, ischemia/reperfusion^[10,11]^, myocardial infarction (MI)^[12]^, or doxorubicin treatment^[13–15]^ [Figure 1]. β-adrenergic receptor stimulation and inflammation may also contribute to cardiomyocyte senescence during aging.

Senescent cardiomyocytes exhibit features of senescence commonly seen in other cell types, including enlarged cell size, DNA damage responses, senescence-associated β-galactosidase (SA-β-gal) activity, the SASP, and up-regulation of p53/p21 or p16/retinoblastoma tumor suppressor protein cell signaling pathways [Figure 1]. For example, phosphorylation of the histone variant H2AX at Ser-139 (γH2AX) is an early cellular response to the induction of DNA double-strand breaks. Thus, γH2AX is a sensitive marker of damaged chromatin in cardiomyocytes. Although the role of the p53/p21 pathway, which mediates cell cycle arrest, remains to be elucidated in terminally differentiated cardiomyocytes, it may be involved in DNA damage responses and the SASP in senescent cardiomyocytes^[16]^. It should be noted that senescent cells are highly heterogeneous and their properties are dynamically altered^[17]^. Thus, senescent cardiomyocytes may consist of multiple cell populations with distinct features. Furthermore, senescent cells in the heart can be either beneficial or detrimental depending on the cell types and conditions in which they are induced. Senescence in cardiac fibroblasts and endothelial cells may affect the heart differently from that in cardiomyocytes (See below). Thus, conducting a deeper characterization of the gene expression profile in senescent cardiomyocytes and other cell types in the heart at the single-cell level is important. Using single-cell RNA sequencing analyses, it may be possible to define a group of senescent cardiomyocytes and predict their function through deep characterization of the gene expression signature^[18]^.

## THE DETRIMENTAL EFFECTS OF SENESCENT CARDIOMYOCYTES IN THE HEART

**Figure 2.**
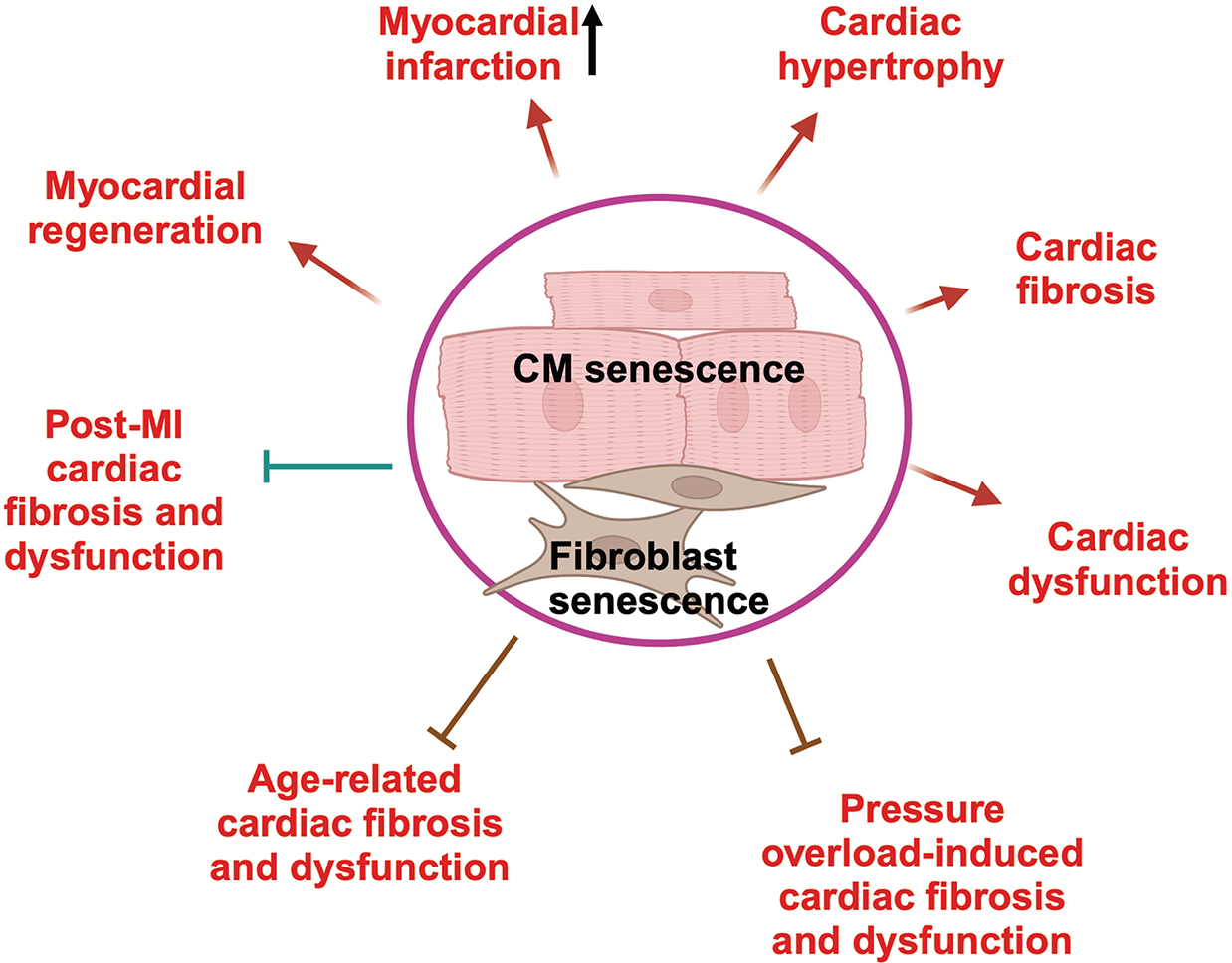
The role of senescent cells in the heart. Senescent cells in the heart have both salutary and detrimental effects on the heart. The detrimental effects of senescent cardiomyocytes include cardiac hypertrophy, cardiac fibrosis, cardiac dysfunction, and larger myocardial infarct size. On the other hand, senescent cardiomyocytes may promote myocardial regeneration. Senescent cardiac fibroblasts may have inhibitory effects on age-related or pressure overload-induced cardiac fibrosis and dysfunction. The senescent cells (cardiomyocytes and fibroblasts together) may attenuate post-myocardial infarction (MI) cardiac fibrosis and dysfunction. The figure was generated with BioRender.com.

There is growing evidence that the accumulation of senescent cardiomyocytes contributes to cardiac dysfunction and adverse remodeling during aging and other pathological conditions [Figure 2]. The proportion of SA-β-gal-positive cardiomyocytes is significantly higher in old mice than in young mice^[8]^, suggesting age-associated accumulation of senescent cardiomyocytes. Aging is accompanied by mechanisms that promote oxidative stress and DNA damage, which promote senescence. Senescent cells secrete SASP factors to recruit immune cells, including macrophages, natural killer (NK) cells, neutrophils, and T lymphocytes, to eliminate themselves. However, senescent cells can also interact with immune cells to block their function and inhibit killing or safe removal through efferocytosis^[19]^. Senescent cells may accumulate during aging and chronic disease conditions, and the accumulated cells can induce deleterious effects by escaping the killing effect of NK cells and suppressing efferocytosis by macrophages^[20]^. The mechanism through which senescent cardiomyocytes escape from the immune surveillance system remains to be clarified^[21]^. A decline in the endogenous mechanisms that eliminate senescent cells could lead to their accumulation and persistent activation of the SASP. Pharmacogenetic clearance of p16-positive cardiomyocytes reduces age-associated myocardial hypertrophy and fibrosis^[7]^. Pharmacological elimination of senescent cells in the heart also decreases the infarct size and increases the ejection fraction five weeks after ischemia/reperfusion^[10]^. Genetic ablation of p16 in cardiomyocytes also decreases the number of senescent cardiomyocytes, improves cardiac function, and decreases fibrosis five weeks after ischemia/reperfusion^[11]^. Doxorubicin (Dox) treatment increases myosin heavy chain-positive (MHC^+^)/p16^INK4a^-positive (p16^+^) cells in the rat left ventricle^[22]^, suggesting that Dox induces cardiomyocyte senescence. Elimination of senescent cells improves cardiac function in the mouse model of Dox-induced cardiomyopathy^[13]^, although whether this is applicable in humans requires further investigation^[23]^. Hutchinson-Gilford progeria syndrome (HGPS) is a premature aging disorder caused by a mutation of the LMNA gene and a truncated lamin A protein, called progerin. Progeria syndrome is accompanied by increases in DNA damage and accelerated senescence in various cell types, including vascular endothelial cells and cardiac fibroblasts, leading to organ dysfunction^[24]^. These results suggest that senescent cells in the heart may promote cardiac dysfunction and provide strong support to the idea of targeting senescent cells in the heart as a modality to treat cardiac dysfunction. To this end, it is important to correctly understand the functional significance of cardiomyocyte senescence. We speculate that senescent cardiomyocytes exert a detrimental effect on the heart through secretion of paracrine factors that induce inflammation in surrounding cells. Although it has been proposed that senescent cardiomyocytes have compromised contractile function^[1]^, how cardiomyocyte senescence or autocrine production of SASP factors, such as IL-6 and TNF-α, affect the contractile function of cardiomyocytes themselves awaits further investigation.

## THE CARDIOPROTECTIVE EFFECTS OF SENESCENT CELLS IN THE HEART

Before we discuss senolysis, it is important to summarize the adaptive and salutary actions of senescence in the heart. Cell senescence can sometimes develop as an adaptive mechanism [Figure 2]. For example, cellular senescence has been reported to have salutary effects during MI^[25,26]^, tissue regeneration^[27,28]^, and pressure overload-induced cardiac fibrosis^[29]^. In the mouse model of MI, senescence marker-, namely p16^INK4a^-, positive cells observed in the border zones were α-actin positive^[25]^, indicating increased cardiomyocyte senescence after MI. Interestingly, AAV9-*Gata4*-shRNA administration significantly attenuated the SASP and aggravated post-MI heart dysfunction^[25]^, suggesting that the premature senescence of cells in the heart, including cardiomyocytes, may play a protective role in the post-MI heart. In the mouse heart with MI, activation of the DNA damage response induces cardiac fibroblast senescence, suppressing the proliferation of non-senescent cardiac fibroblasts and attenuating the progression of cardiac fibrosis^[26]^. In mice that undergo visceral adipose tissue removal and osteopontin knockout mice, a strong induction of senescence in cardiac fibroblasts results in protection against age-related cardiac fibrosis and dysfunction^[30]^. In neonatal mouse hearts, apical resection-induced CCN1 secretion from cardiomyocytes induces fibroblast senescence, which in turn promotes myocardial regeneration by enhancing cardiomyocyte proliferation and reducing cardiac fibrosis^[27]^. Similarly, apical resection of the neonatal mouse heart induces transient high levels of senescence in fibroblasts, marked by senescence-associated β-galactosidase staining^[28]^, although the specific role of fibroblast senescence in myocardial regeneration remains to be shown. The transient kinetics of induction and clearance of senescent cells in this model might suggest that regenerative senescence is beneficial in promoting tissue repair. In response to pressure overload, myofibroblasts were the predominant cell population undergoing premature senescence in the heart^[29]^. Inactivation of the premature senescence program resulted in aggravated fibrosis and impaired cardiac function after transverse aortic constriction^[29]^. Conversely, inducing premature senescence of cardiac fibroblasts by adeno-associated virus serotype 9 gene transfer-mediated cardiac-specific expression of CCN1 (CYR61) resulted in about 50% reduction of perivascular fibrosis after transverse aortic constriction^[29]^. These results suggest that the function of senescent cells is not only context-dependent but also cell type-dependent. It should be noted that senescent fibroblasts are not always protective. For example, senescence of fibroblasts induced by p53 deficiency promotes fibroblast collagen deposition *in vitro* and myocardial fibrosis *in vivo*^[31]^. The discrepancy among different studies regarding the role of senescent fibroblasts in MI highlights the complexity of senescence.

## THE SASP IN SENESCENT CARDIOMYOCYTES

Senescent cells secrete inflammatory cytokines, chemokines, growth factors, proteases, and insoluble proteins/extracellular matrix components, a phenomenon termed the SASP. The factors produced by the SASP have both salutary and detrimental effects. In the case of cancer, the SASP of senescent cells has both tumor suppressor and tumorigenic properties (reviewed in^[32]^). The salutary effect of the SASP was observed in skeletal muscle regeneration following acute injury, in which senescent cells facilitate cellular plasticity via SASP factors, particularly IL6^[33]^. During skin wound healing, senescent fibroblasts and endothelial cells secrete platelet-derived growth factor-AA, which induces myofibroblast differentiation to accelerate wound closure^[34]^. On the other hand, the SASP factors activate feedforward mechanisms, causing amplification and spreading of senescence^[35]^. The SASP factors also induce inflammation. In aged mouse hearts, activation of NFκB increases senescence and enhances the SASP, including secretion of proinflammatory cytokines IL1β and IL6, thereby increasing sterile inflammation^[36]^. On the other hand, cardiac-specific inhibition of Ca^2+^/calmodulin-dependent protein kinase II decreases the NFκB signaling and the levels of IL1β and IL6, thereby alleviating chronic sterile inflammation and aging-associated cardiomyopathy in mice^[36]^.

**Figure 3.**
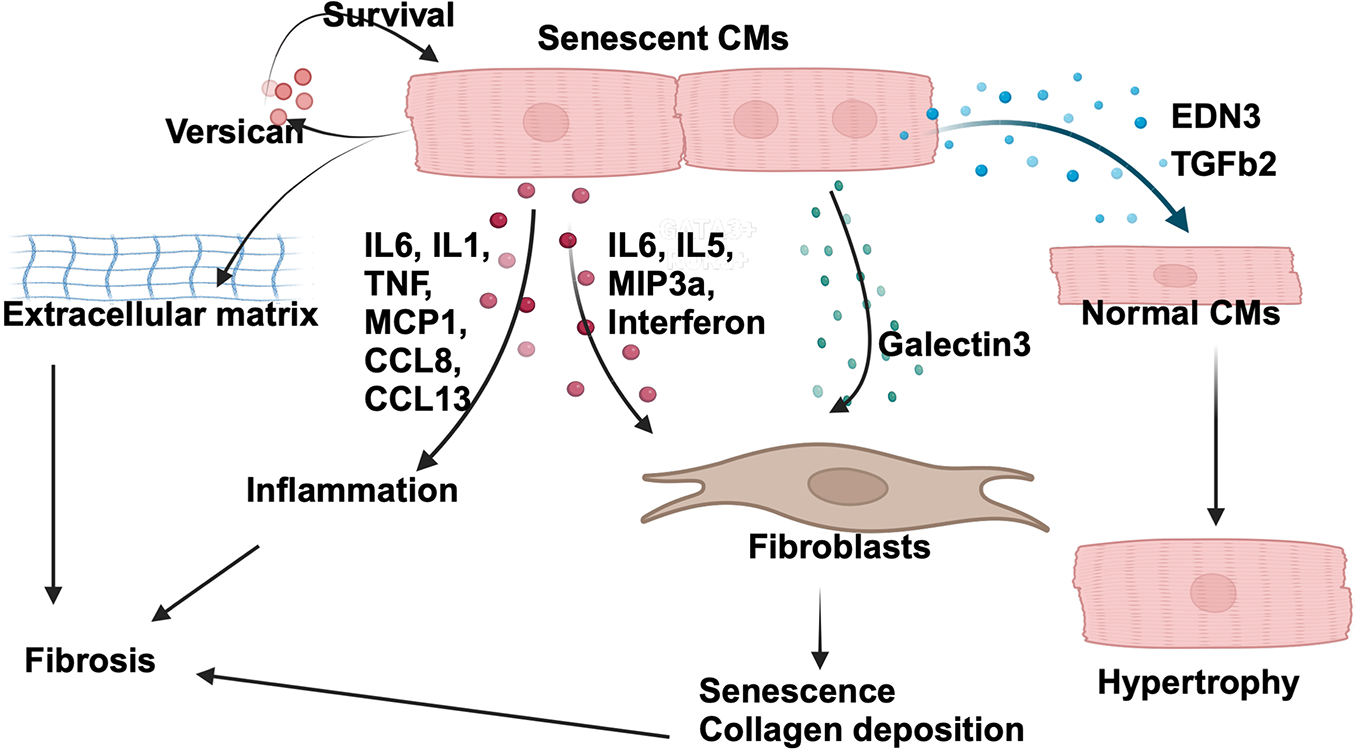
The senescence-associated secretory phenotype (SASP) of senescent cardiomyocytes. Senescent cardiomyocytes secrete an array of factors that affect the surrounding cells and maintain their senescent state. The figure was generated with BioRender.com.

Autocrine/paracrine factors produced in senescent cardiomyocytes include proinflammatory cytokines (*Tnf*, *Il-6*, *Il-1β*, *Il-8* and *Mcp-1/Ccl2*), extracellular matrix factors (*Col4a4, Col4a3, Col9a2*, and *Lamb3*), chemokines (*Ccl8* and *Cxcl13*), cytokine receptors (*Tnfrsf9*, *Ifnlr1*, *Il18rap*, and *Il2ra*), enzymes (*Adcy9* and *Prkcz*), and growth factors (*Egf* and *Fgf*)^[36]^. Importantly, the specific factors produced by senescent cardiomyocytes appear to be context-dependent. Cardiomyocytes from aged mice exhibit higher levels of several senescence markers and produce *Edn3*, *Tgfb2*, and *Gdf15*, but not typical proinflammatory cytokines^[7]^. *Edn3* and *Tgfb2*, encoding Endothelin 3 and TGF-β2, respectively, may induce cardiac hypertrophy^[37]^, whereas GDF15 induces cellular senescence^[38]^ and inflammation^[39]^. Cardiomyocytes isolated from cardiac-specific transmembrane protein 43 knockout mice exhibit increases in the DNA damage response and SASP factors, including *Lgals3, Vcan, Tgfb2*, and *Gdf15*^[40]^. *Lgals3*, encoding Galectin-3, a soluble β-galactoside-binding protein, promotes cardiac fibroblast proliferation, collagen deposition, and ventricular dysfunction (reviewed in^[41]^). *Vcan*, encoding Versican, activates integrin β1-ERK1/2 and Akt signaling to promote survival and proliferation^[42]^. Overall, these factors produced by senescent cardiomyocytes may play a prominent role in the development and maintenance of senescence in cardiomyocytes and the whole heart [Figure 3 and Table 1]. In general, transcription of SASP factors is regulated by NF-κB, C/EBP-β, p53 and Rb^[43]^. Since the SASP is cell type- and stimulus-specific, we speculate the identity of responsible transcription factors varies in a context-dependent manner.

## INTERACTION WITH NON-MYOCYTES

The paracrine effect of SASP factors produced in cardiomyocytes on other cell types in the heart appears complex. SASP factors may spread senescence to surrounding cells, which in turn induces cell type-specific actions. For example, the atypical SASP factors produced in aging cardiomyocytes, namely *Edn3*, *Tgfb2*, and *Gdf15*, increase α-smooth muscle actin, an indicator of myofibroblast activation, but induce senescence in fibroblasts^[7]^. Deletion of p16 in cardiomyocytes not only decreases the number of senescent cardiomyocytes but also reduces the number of senescent interstitial cells (fibroblasts in particular), possibly through decreased secretion of SASP factors such as IL-6, interferon γ, IL-5, and macrophage inflammatory protein 3α from cardiomyocytes^[11]^. Senescent fibroblasts promote fibroblast collagen deposition *in vitro* and myocardial fibrosis *in vivo*^[31]^. Furthermore, induction of senescence in cardiac fibroblasts may induce the SASP^[44]^, which may induce inflammation and inhibit reparative fibrosis^[31]^. Senescence of endothelial cells contributes to the development of heart failure with preserved ejection fraction^[45]^. Thus, senescent cardiomyocytes could exert adverse effects on the heart by influencing non-myocytes via the SASP factors and induction of senescence. However, as we discussed earlier, senescence in cardiac fibroblasts and other non-myocyte populations is also involved in tissue remodeling and regeneration in a context-dependent manner^[27,28]^. Thus, the molecular identities of the SASP factors mediating either detrimental or salutary effects in the heart and the mechanisms through which their expression is regulated require further investigation.

## SENOLYSIS

Senolysis is the selective elimination of senescent cells. The concept of senolysis was developed based on the inverse association between the senescent cell burden in multiple rodent organs/tissues and health span. The expression of *p16*^*INK4a*^ and *Arf* is markedly increased in the tissues of aged mice, whereas interventions to delay aging in many organisms, such as caloric restriction, delay the accumulation of p16^INK4a^- and SA-β-gal-positive cells in mice and extend their health span^[46]^. Thus, there is a great interest in finding strategies to specifically eliminate senescent cells but not non-senescent cells, i.e., senolysis^[47]^. Growing evidence supports the rationale of senolysis and its anti-aging effects. Since aging is a major risk factor for heart disease, senolysis could represent a promising intervention for the heart with senescent cardiomyocytes. Importantly, however, unlike regenerative and proliferative cells, cardiomyocytes are terminally differentiated. Thus, cardiomyocytes may not be replenished after senolysis. This raises the question of whether senolysis improves cardiac function despite the loss of cardiomyocytes. Although there have been reports suggesting that removal of cardiomyocytes by senolytics has salutary effects, thorough investigation into the mechanisms of cardiomyocyte senescence, especially the mechanisms through which cardiomyocytes develop and maintain the senescent state, is critical to identify or develop strategies for “safe” senolysis. Senolysis is largely categorized into two groups according to the mechanism of action: one targets signaling mechanisms uniquely activated in senescent cells, whereas the other selectively directs cytotoxic mechanisms to senescent cells.

**Figure 4.**
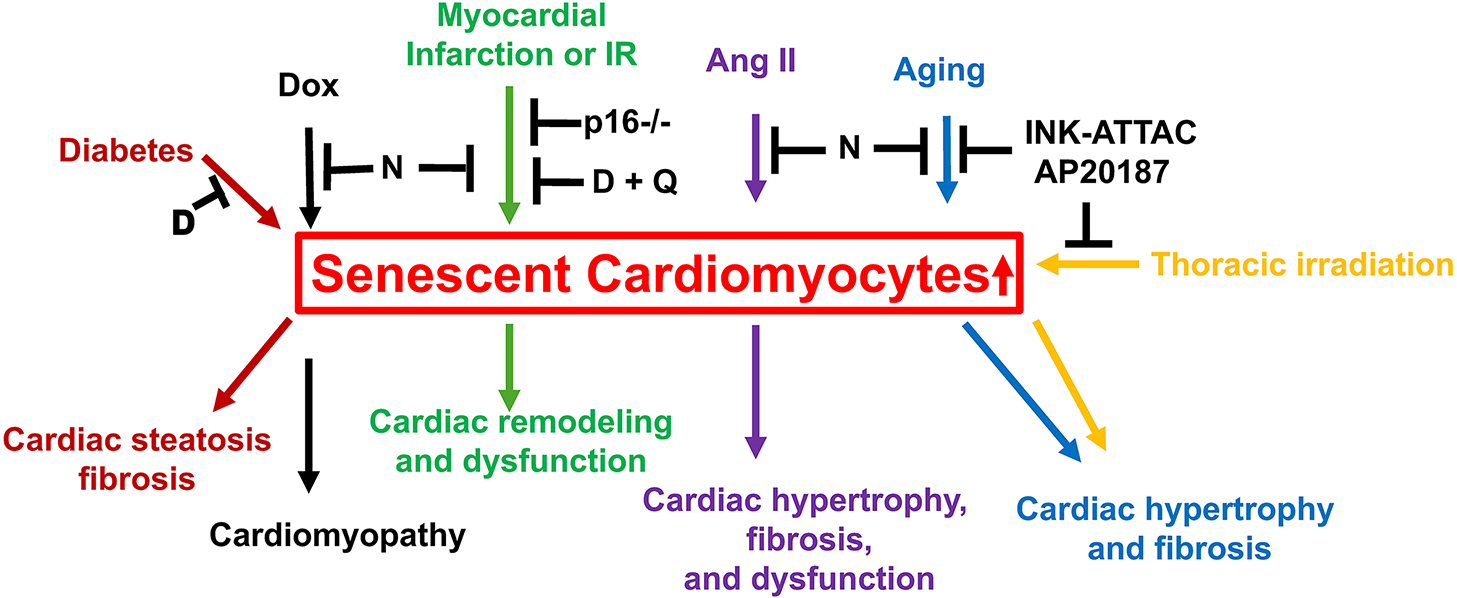
Induction of senescence in cardiomyocytes and the effectiveness of senolysis under these conditions. Stressors in the heart, including diabetes, doxorubicin (Dox), myocardial infarction or ischemia/reperfusion (IR), angiotensin II (Ang II), aging, and thoracic irradiation, induce senescence in cardiomyocytes. Senolytic drugs, including Navitoclax (N), Dasatinib (D), and Quercetin (Q), or induction of senolysis, via p16 deletion or INK-ATTAC plus AP20187, reduce the number of senescent cells, including cardiomyocytes, in the heart, thereby attenuating cardiac hypertrophy, fibrosis, and dysfunction. Specific stressors and their resultant consequences are indicated with the same color.

The development/identification of senolytic drugs has been based on the presence of unique features in senescent cells. Senescent cells resist cell death induced by apoptotic stimuli, such as serum withdrawal^[48]^, indicating the presence of enhanced pro-survival and/or increased anti-apoptotic defenses (for example, Bcl-2). Senescent cells have the ability to prevent their own death through activation of anti-apoptotic networks orchestrated by ephrins (Eph) B1, EphB-3, phosphatidylinositol-4,5-bisphosphate 3-kinase delta catalytic subunit (PI3KCD), cyclin-dependent kinase inhibitor 1A (CDKN1A/p21), plasminogen activator inhibitor-2 (PAI-2), Bcl-xL, and MCL-1^[49]^. The phenotype associated with cellular senescence is highly heterogeneous and complex, and, therefore, there are no universal markers of cellular senescence. This limits the development and evaluation of senolytics that selectively target senescent cells without off-target side effects. In attempting to solve the problem, strategies that more specifically target senescent cells have been developed and tested. To date, there are several chemicals/drugs that have senolytic properties, such as ABT263, A1331852, A1155463, UX1325, Dasatinib, Quercetin, Fisetin, Ouabain, and Digoxin. However, only a few studies have been done on cardiomyocytes thus far [Figure 4].

### Dasatinib and quercetin

Dasatinib is an inhibitor of receptor tyrosine kinases, including ephrin type-A and type-B receptor kinases^[50]^, and is known to interfere with EphB-dependent suppression of apoptosis^[51]^. Quercetin is a naturally occurring flavonoid that interferes with PI3K/Akt stimulation and AMP-dependent protein kinase activation, thereby inhibiting mTOR signaling^[52]^. Quercetin also has strong anti-inflammatory effects. Dasatinib and Quercetin can each induce the death of senescent cells when used separately, but when used together, they exhibit synergistic effects and a broader spectrum of senolysis^[49]^. Dasatinib decreases the number of senescent cells in the heart and reduces cardiac steatosis and fibrosis in obese, type 2 diabetic mice^[53]^. The combination of Dasatinib and Quercetin decreases the number of senescent cardiomyocytes and non-myocytes in aged female mice after MI^[12]^, and ameliorates cardiac remodeling and dysfunction. It should be noted that Dasatinib, used as an anticancer therapy, is cardiotoxic, causing cardiac dysfunction and heart failure, possibly due to the death of non-senescent cardiomyocytes^[54]^.

### Navitoclax (ABT-263)

Navitoclax (ABT-263) is a potent Bcl-2 family protein inhibitor that targets Bcl-2, Bcl-xL, and Bcl-W. Navitoclax can selectively kill senescent cells, at least in irradiation-induced senescent cells, by simultaneously inhibiting Bcl-2 and Bcl-xL^[55]^. The specific elimination of senescent cardiomyocytes by Navitoclax has been observed in HL-1 cardiomyocytes treated with doxorubicin^[13]^. Navitoclax decreases senescence and cardiotoxicity and restores cardiac function in mice with doxorubicin-induced cardiomyopathy^[13]^. It also eliminates angiotensin II-induced senescent cardiomyocytes and cardiac fibroblasts and improves cardiac dysfunction, attenuates cardiac hypertrophy and fibrosis, and alleviates the inflammatory reaction in an angiotensin II-induced heart failure mouse model^[56]^. Navitoclax eliminates senescent cells (including cardiomyocytes) in the peri-infarct zone and improves cardiac function following cardiac ischemia-reperfusion injury^[10]^. Navitoclax also removes senescent cardiomyocytes, ameliorates profibrotic protein expression in aged mice, and attenuates adverse myocardial remodeling and diastolic dysfunction, improving overall survival following MI^[57]^. In aged wild-type mice, Navitoclax reduces the number of cardiomyocytes with telomere-associated foci (i.e., senescent cardiomyocytes) and significantly reduces hypertrophy and fibrosis^[7]^.

It should be noted, however, that Navitoclax also exhibits adverse effects, such as thrombocytopenia and neutropenia^[58]^, possibly due to its effects on non-senescent cells. In order to target Navitoclax specifically to senescent cells, strategies using nanoparticles as carriers have been developed, allowing its controlled and selective release in senescent cells. For example, nanoparticles loaded with Navitoclax and coated with a hexagalactosaccharide, galactan, were shown to more potently and specifically remove senescent cardiomyocytes^[13]^. This strategy utilizes the prodrug concept and the fact that senescent cells have high lysosomal β-gal activity. Although Navitoclax coated with galactan has no pharmacological activity, when it reaches senescent cells, the lysosomal β-gal induces the hydrolysis of the cap and releases Navitoclax only into senescent cells. Another example is the use of matrix metalloproteinase-3 (which is increased in senescent cells)-gated Navitoclax-loaded nanodevices to induce senescent cells’ death while preserving proliferating cells’ viability^[59]^.

### Selective targeting of the cytotoxic effect to senescent cells

Although Dasatinib, Quercetin, and Navitoclax are designed to target unique signaling mechanisms in senescent cells, they have off-target effects. An alternative strategy is to direct a cytotoxic mechanism specifically to senescent cells, using genetic methods in conjunction with gene delivery methods or using immunological interventions. The gene delivery approach utilizes senescent cell-specific expression of transgenes encoding cell death-inducing factors, directed by the p16- or p21-promoter^[60,61]^, whereas immunological interventions involve selective recognition and killing of senescent cells by immune cells and antibodies through recognition of specific cell surface antigens on senescent cells^[59,60]^. Although identifying unique cell surface antigens for the latter approach is extremely time-consuming, it could minimize off-target effects.

#### INK-ATTAC model

By using a biomarker for senescence, p16^INK4a^, a novel transgenic mouse model, INK-ATTAC, has been generated for inducible elimination of p16^INK4a^-positive senescent cells^[62]^. These mice contain a transgene coding for a membrane-bound myristoylated FK506-binding protein - caspase-8 fusion protein (the suicide signal) under the control of a promoter from the *Cdkn2a(Ink4a)* gene. Administration of a synthetic drug, AP20187, induces dimerization and activation of caspase-8 and, ultimately, apoptosis in p16^INK4A^-expressing cells^[62]^. In aged INK-ATTAC mice or in mice subjected to thoracic irradiation, administration of AP20187 reduces senescent cardiomyocytes and attenuates age-associated cardiac hypertrophy and fibrosis^[7]^.

#### p16-LOX-ATTAC model

This mouse model was developed in order to achieve p16^INK4a^-mediated expression of ATTAC in a tissue-specific manner^[63]^. When crossed with tissue-specific *Cre* lines, the Cre deletes the loxP-flanked gene segments (EGFP 3xSTOP) in these mice and allows cell type-specific elimination of senescent cells expressing high levels of p16.

#### p16–3MR model

p16–3MR mice carry a trimodal reporter protein (3MR) containing Renilla luciferase, monomeric red fluorescent protein (mRFP), and herpes simplex virus thymidine kinase (HSV-TK) under the control of the p16^INK4A^ promoter, which allows for selective genetic clearance of excess p16^INK4A^-positive senescent cells by administering the antiviral agent Ganciclovir^[34]^. In mouse models utilizing the p16 or p21 promoter for senescent cell-specific gene manipulation, several versions of the p16 and p21 promoters with increased efficacy have been reported^[60,61]^.

#### Urokinase-type plasminogen activator receptor-specific chimeric antigen receptor T cells

Chimeric antigen receptor (CAR) T cells are T cells that are genetically engineered to express CARs, which enable the T cells to target cells with specific antigens. CARs consist of an extracellular antigen-recognition domain, which permits the recognition of a specific antigen by a T cell, and an intracellular signaling domain, which stimulates T-cell proliferation, cytolysis, and cytokine secretion to eliminate the target cell. The urokinase-type plasminogen activator receptor (uPAR), a cell-surface protein, is broadly induced during senescence, and uPAR-specific CAR T cells efficiently ablate senescent cells *in vitro* and *in vivo*^[64]^. Treatment with uPAR-targeted CAR T cells significantly prolongs survival without eliciting signs of toxicity in mice with lung adenocarcinoma that are treated with a senescence-inducing combination of drugs. The high senolytic activity of uPAR CAR T cells is able to induce an efficient reduction in liver fibrosis and a sustained resolution of liver fibrosis of different etiologies^[64]^.

#### Senolytic vaccination

Glycoprotein nonmetastatic melanoma protein B (GPNMB), a molecule with a transmembrane domain, was found to be enriched in senescent vascular endothelial cells (seno-antigen)^[65]^. Furthermore, genetic ablation of GPNMB-positive cells attenuates senescence in adipose tissue. Immunization of mice against GPNMB reduces the number of GPNMB-positive cells^[65]^. In addition, the senolytic vaccination improves normal and pathological phenotypes associated with aging in mice and extends the lifespan of male progeroid mice^[65]^. These results indicate that vaccination against seno-antigens (i.e., senolytic vaccination) could be a potential strategy for senolytic therapies.

## SUMMARY

In this article, we discussed potential applications of senolysis as a modality to treat cardiac conditions in which cell senescence is observed in the heart. Removal of senescent cardiomyocytes as a therapeutic strategy for heart failure is counter-intuitive because eliminating adult cardiomyocytes that rarely regenerate would result in a reduction of the total number of contracting cardiomyocytes. However, since even a small number of senescent cardiomyocytes may negatively affect many nearby cardiomyocytes, the removal of senescent cardiomyocytes could be salutary. The basic strategy of senolysis in the heart is similar to that of cancer chemotherapy but opposite from that of conventional heart failure treatment, which aims to promote the survival of cardiomyocytes. Senolytic therapy in the heart adopts the same general concept as chemotherapy since both target a unique mechanism essential for the survival of cancer/senescent cardiomyocytes and/or selectively deliver the cytotoxic mechanisms to them. To achieve these goals, a detailed characterization of senescent cardiomyocytes distinguishing them from non-senescent cardiomyocytes and a thorough investigation into the mechanisms through which cardiomyocytes develop senescence and/or maintain the senescent state are needed. For example, it is important to identify the signaling mechanisms and metabolic pathways that are more strongly activated in senescent cardiomyocytes than in non-cardiomyocytes, allowing them to survive even in a stressful environment. It is generally believed that cardiomyocytes in the aged heart undergo functional decline and mitochondrial dysfunction^[1]^. However, whether these features are also observed in senescent cardiomyocytes needs to be tested, since senescent cells are generally metabolically more active than non-senescent cells. It is possible that senescent cardiomyocytes may be distinguished from non-senescent cardiomyocytes based on mitochondrial function and metabolism. Another important feature of senescent cells is their ability to propagate senescence through paracrine mechanisms^[66]^. For example, SASP signals produced by senescent cardiomyocytes induce senescence in fibroblasts^[7]^. Thus, it is also important to decode the complex cell-to-cell interaction mechanisms within the heart in the presence of senescent cardiomyocytes. Research into cardiomyocyte senescence is still at a primitive stage and many fundamental questions remain, as summarized in Box 1. Addressing these questions may lead to the development of novel interventions to achieve safe treatment for the heart through modulation of cellular senescence.

## Figures and Tables

**Table 1.** The SASP factors produced by cardiomyocytes in various cardiac conditions

Cardiac conditions	SASP factors	Cell sources	References

Aging	*Edn3, Tgfb2, Gdf15*	Cardiomyocytes	[1]
Aging	*Tnf, IL6, IL1β, IL8, Mcp1, Col1a, Col4a3, Col9a2, Lamb3, Ccl8, Cxcl13, Tnfrsf9, Infnlr, Il18rap, Il2ra, Adcy9, Prkcz, Egf, and Fgf*	Cardiomyocytes	[36]
Ischemia/reperfusion	*IL6, IL5, IFNγ, MIP3a*	Cardiomyocytes	[11]
Arrhythmogenic cardiomyopathy	*Lgals3, Vcan, Tgfb2, Gdf15*	Cardiomyocytes	[40]

## Data Availability

Not applicable.

## Box 1. Outstanding issues in the study of cardiac senescence

### Identifying senescent cells *in vivo*:

The lack of selective markers for cellular senescence and the heterogeneous and dynamic nature of senescent cells make identification and characterization of senescent cells challenging *in vivo*

### Cell type-specific actions of senescent cells:

The function of senescent cells may differ among various cell types, such as cardiomyocytes *vs*. cardiac fibroblasts, which complicates pharmacological interventions in the absence of cell type-specific targeting

### Properties of senescent cardiomyocytes:

Although senescent cells may cause organ dysfunction and aging through the SASP, they are also involved in organ development and repair processes. The molecular identity of the SASP factors involved in each process needs to be clarified. Senescent cells are generally cell death-resistant and metabolically active. It remains unclear how mitochondrial function is maintained to support the SASP in senescent cardiomyocytes. How changes in contractility and metabolism in senescent cardiomyocytes affect the function of the heart is not fully understood

### Mechanism of spreading:

Cellular senescence is spread and amplified through autocrine/paracrine mechanisms. The relevant cell-to-cell interactions need to be better understood

### Clearance of senescent cardiomyocytes:

Insufficient clearance of senescent cells leads to the accumulation of senescent cells and stimulation of chronic inflammation. How the immune surveillance system is affected by senescent cells, as well as stress, needs to be better understood

### Senescent cell-specific targeting and off-target effects of senolytics:

The current modalities of senolysis may inadvertently also target non-senescent cells. Signaling mechanisms and/or cell surface antigens uniquely altered and involved in the survival and/or SASP in senescent cells need to be identified. Novel interventions allowing senescent cell-specific targeting need to be developed
